# Socioeconomic Inequalities and Obesity in South Africa—A Decomposition Analysis

**DOI:** 10.3390/ijerph18179181

**Published:** 2021-08-31

**Authors:** Eva Goetjes, Milena Pavlova, Charles Hongoro, Wim Groot

**Affiliations:** 1 CINCH Health Economics Research Center, Faculty of Business Administration and Economics, University of Duisburg-Essen, Berliner Platz 6–8, 45127 Essen, Germany; 2 Department of Health Services Research, CAPHRI, Maastricht University Medical Centre, Faculty of Health, Medicine and Life Sciences, Maastricht University, P.O. Box 616, 6200 MD Maastricht, The Netherlands; m.pavlova@maastrichtuniversity.nl (M.P.); w.groot@maastrichtuniversity.nl (W.G.); 3 Peace and Sustainable Security (PaSS), Developmental, Capable and Ethical State Division, Human Sciences Research Council, 134 Pretorius Street, Private Bag X41, Pretoria 0001, South Africa; CHongoro@hsrc.ac.za; 4 School of Health Systems and Public Health, University of Pretoria, Private Bag X323, Pretoria 0001, South Africa

**Keywords:** equity, socioeconomic inequalities, obesity, Erreyger concentration index, decomposition analysis

## Abstract

Background: Prior evidence shows that inequalities are related to overweight and obesity in South Africa. Using data from a recent national study, we examine the socioeconomic inequalities associated with obesity in South Africa and the factors associated with it. Methods: We use quantitative data from the South African National Health and Nutrition Examination Survey (SANHANES-1) carried out in 2012. We estimate the concentration index (CI) to identify inequalities and decompose the CI to explore the determinants of these inequalities. Results: We confirm the existence of pro-rich inequalities associated with obesity in South Africa. The inequalities among males are larger (CI of 0.16) than among women (CI of 0.09), though more women are obese than men. Marriage increases the risk of obesity for women and men, while smoking decreases the risk of obesity among men significantly. Higher education is associated with lower inequalities among females. Conclusions: We recommend policies to focus on promoting a healthy lifestyle, including the individual’s perception of a healthy body size and image, especially among women.

## 1. Introduction

In 2015, worldwide, roughly 11% of men and 15% of women above the age of 18 were obese [1]. The World Health Organization (WHO) defines overweight and obesity as “abnormal or excessive fat accumulation that impairs health” and classifies persons in these groups based on their body mass index (BMI). BMI of 25 kg/m^2^ or higher represents ‘overweight’ while ‘obesity’ is defined as a BMI equal to 30 kg/m^2^ or higher [2]. Overweight and obesity are important risk factors for noncommunicable diseases (NCD), leading to higher mortality [3]. A 30% increase in mortality is reported per 5 kg/m^2^ higher BMI and is directly linked to the incidence of cardiovascular diseases, respiratory diseases, and cancer [2,4]. There is a strong correlation between diabetes mellitus type 2 and overweight or obesity, as it was found that 75% of diabetes patients are overweight or obese [4,5]. The increasing prevalence of overweight and obesity not only increases mortality rates but increases the demand for scarce healthcare resources [6]. Severe obesity has been shown to increase healthcare costs by up to 23% [7].

Health inequalities refer to the differences and disparities in the health status of individuals and different groups of individuals [8]. These disparities derive from social, cultural, demographic, and economic factors and backgrounds of individuals [9,10]. It is argued that inequalities are unfair and unjust, that they affect everyone, and that the reduction of health inequalities is cost-effective [11,12]. Studies provide evidence that poor health is related to an individual’s education, income, and occupation and therefore is more prevalent in less affluent parts of society [13,14]. This has a direct influence on these less affluent populations and an indirect influence on their better-off counterparts as well. An example of this indirect influence is obesity. Obesity has been shown to lead to poor health, leading to rising cases of illnesses, which can cause a higher mortality rate besides an increasing need for scarce resources, resulting in a negative influence on a country’s health expenditure [6].

The gravity of inequalities in health as well as socioeconomic or sociodemographic inequalities is rather clear when one looks at the case of South Africa. Due to the history of colonialization and the devastating Apartheid regime, the country is still one of the most unjust in the world [15]. This is reflected, for instance, by large inequalities across socioeconomic as well as racial groups, measurable by indicators such as higher poverty and unemployment (Human Development), strong variations in educational levels, health outcomes, and access to health care [15,16,17,18,19].

Various studies have examined the relationship between obesity and socioeconomic factors and have provided evidence of socioeconomic inequalities in association with adult obesity [20,21,22,23,24,25,26,27]. These inequalities associated with obesity are linked to factors, like age and gender, but also socioeconomic factors, such as income, education, or inequalities related to sociodemographics [22].

Overweight and obesity affect populations worldwide, including people in low- and middle-income countries. Already in 1997, the WHO foretold that overweight and obesity would become one of the leading health problems in low- and middle-income countries [28]. Since the economic growth in these countries leads to a wealthier middle class and thereby to a shift in nutrition and physical activity that increases the risk of overweight and obesity [29].

This paper focuses on inequalities associated with obesity in South Africa. Previous studies have used the 2008 Africa National Income Dynamic Survey to calculate inequalities in obesity. In these studies, it was found that wealthier men were more likely to be overweight or obese, with similar findings for women. Overall, a positive relationship between body weight and socioeconomic status (SES) was found, i.e., the higher the SES, the higher the rates of obesity tended to be in South Africa [21]. It is, however, unclear whether this trend has persisted in more recent years.

We aim to examine socioeconomic inequalities associated with obesity in South Africa and the socioeconomic factors that are associated with these inequalities by using data from the South African National Health and Nutrition Examination Survey (SANHANES-1) carried out in 2012 [30]. This is the most recent data source of representative household data available in South Africa. The paper is of interest to decision-makers and researchers in South Africa as well as in other low- and middle-income countries undergoing a process of epidemiological transition from infectious to NCD.

## 2. Methods

This study used the SANHANES-1 dataset [30]. This cross-sectional survey was carried out in 2012 by the Human Sciences Research Council in collaboration with the Medical Research Council, to collect information on the health and nutritional status of South Africa’s population. The dataset was provided to us after it was anonymized.

### 2.1. Sampling

A multi-stage disproportionate, stratified cluster sampling approach was applied to obtain the survey sample [30]. In particular, 500 enumerator areas (EAs) were selected based on population census data. The selection of EAs was stratified by province and locality type, as well as by race in the urban areas. In urban areas dominated by one race group, an over-allocation of EAs occurred to ensure sufficient observations of smaller race groups. In total, 20 visiting points (households) were randomly selected from each EA, yielding an overall sample of 10,000 households. Of these households, 8168 households were contacted. The rest of the households either had an invalid address or the house was abandoned. In total, 6306 (77.2%) households agreed to participate and were interviewed. That led to 25,532 participants (members of the surveyed households) who completed the questionnaire and were invited to participate in the subsequent clinical examination.

### 2.2. Data Collection

The data were collected by 67 trained fieldwork teams distributed throughout the nine provinces. Each team consisted of two sub-teams: one sub-team for the administration of the questionnaires and another mobile (on wheels) clinic sub-team for the examination. The teams were coordinated by four provincial directors and 15 provincial coordinators. The data collectors within each team received 6–10 days of training [30].

The questionnaire consisted of three components:

1. A household questionnaire was completed by the head of the household; key variables included: demographic data, food security, alcohol, health insurance, housing, household goods and services, accessibility to services, and cost of living;
2. An adult questionnaire completed by all persons 15 years of age or older; key variables included: biographic details, NCDs, tuberculosis, nutrition and weight management, perceptions of general health and health care utilization;
3. A child questionnaire completed by either parents or legal guardians of children 0–14 years of age; key variables included: biographic details of the child, parent/guardian’s details, birth registration, breastfeeding, early childhood development, childhood illness and treatment, road to health/clinic card/booklet, HIV, disability, as well as diet, nutrition and weight management.

The subsequent clinical examination included a physician’s examination, blood analyses, cardiovascular fitness test, measurement of weight, height, waist and hip circumference, as well as blood pressure and pulse rate examination. The respondents could voluntarily choose to participate in the clinical examination of this study. As a ‘clinic on wheels’ was not possible, other suitable alternatives were used. This was either in the form of clinic settings in as close as possible proximity to the participant’s home or in case of very rural settings, in the form of a clinical team coming to the participant’s home. Less than half of the eligible individuals, so individuals who had agreed to be interviewed, participated in the clinical examination. The response rate varied around 40% over different age categories, gender, and locations. However, the lowest response rates were mentioned for the age category of 25–34 years old and Caucasian individuals [30].

To correct for possible self-selection biases due to the disproportionate stratified sampling procedure and to adjust for nonresponse, sample weights provided in the SANHANES-1 dataset were used in all analyses. As indicated in the SANHANES report, demographic, physical examination, and clinical examination information were used in order to calculate individual sample weights at each responding level (questionnaire, physical and clinical examination). These individual sample weights were also adjusted for physical examination non-response and clinical examination non-response [30]. Overall, the SANHANES-1 dataset was assessed as reliable and generalizable for the larger population of South Africa [30].

### 2.3. Study Sample

Our study focused on socioeconomic inequalities associated with obesity in adolescents, young adults, and adults. Thus, only the results of participants from 15 years of age onwards were included in our analysis. This included 16,780 participants in total. Not all of these participants completed the clinical examination. Of those eligible, only 43.6% consented to physical examinations, like measurement of weight and height. Therefore, the BMI’s of 7259 participants were computed. We excluded BMI outliers (below 10 kg/m^2^ or above 70 kg/m^2^ [30]) as well as participants younger than 15 or without a gender indication. Thus, we were able to include 7068 participants in the analysis.

### 2.4. Variables

In our study, the key health status variable was ‘obesity’ measured as a binary variable based on the individual BMI calculated from the individual’s weight in kilograms divided by the squared height in meters. A BMI equal to or higher than 30 kg/m^2^ represented ‘obesity’ (‘yes = 1’ and ‘no = 0’). Additional health indicators that could directly influence weight, such as unhealthy diet, physical inactivity, and smoking, were included as control variables. Specifically, the diet of an individual was categorized as “unhealthy” (‘yes = 1’ and ‘no = 0’) if the fruit and vegetable intake was less than one portion a day [31], smoking was measured as ‘ever smoked’ (‘yes = 1’ and ‘no = 0’). Physical inactivity was registered through a cardiovascular fitness test when the pulse rate was lower than the optimal gender-dependent pulse rate of the related age group. Participants were then categorized as “physically inactive” (‘yes = 1’ and ‘no = 0’). This cardiovascular fitness test and the categorization were based on the guidelines of the Canadian Public Health Association Project [30,32].

Another key variable in our study was the wealth index and the corresponding wealth quintiles, constructed by applying Multiple Correspondence Analysis to the household survey data of SANHANES-1 [33]. Therefore, 16 variables, including housing type, water and sanitation services, and asset ownership, were used. The percentage inertia explained by the first dimension is approximately 90%. In addition, sociodemographic variables such as age group, gender, race, education, employment status, and civil status were also included in the analysis.

### 2.5. Data Analysis

To examine the inequalities associated with obesity prevalence, we used the concentration index (CI) [34]. The CI refers to the degree of deviation from the line of perfect equality of obesity among wealthy groups (CI = 0). The deviation from this perfect equality could occur in one of the following directions: higher prevalence of obesity among the more affluent population (CI > 0) or higher prevalence among the less affluent population (CI < 0). Specifically, we used the corrected CI (Erreygers CCI) to address the shortcoming of the classical CI estimates. The subsequent decomposition of the CI enabled us to identify the contribution of SES factors to the inequality in obesity prevalence [27].

The decomposition analysis of a binary variable can be done with a linear probability model (LPM), non-linear logit model, or generalized linear model (GLM). Different approaches are used in former studies; for instance, Hossain et al. [35] conducted a decomposition analysis of socioeconomic inequalities in infant mortality in Iran using non-linear logit model, Alaba and Chola [21] used LPM for their decomposition analysis of obesity, whilst Yiengprugsawan et al. [36] argue, that GLM provides best results of decomposition estimation of a CI. This, like the GLM estimates the average of the individual effect of the independent variable rather than the overall average effect used by the LPM. Additionally, the results using GLM do not vary according to the reference group and provide valid coefficient estimates. Hence, in our study, a GLM approach was used to examine the socioeconomic inequalities in obesity. The statistical analysis of the data was done using the Stata Statistical Software: Release 15 (StataCorp LLC, College Station, TX, USA). In all analyses, we used sample weights.

### 2.6. Ethics Considerations

The SANHANES-1 survey was reviewed by the Research Ethics Committee of the Human Science Research Council and was approved by that committee (REC 6/16/11/11) [30]. The confidentiality of our analysis was assured since the data we received were already anonymized. Thus, we had no access to personal details and were unable to draw lines between a given response and a specific respondent [30,37].

## 3. Results

Table 1 provides an overview of the weighted sociodemographic variables of the study sample by gender. In total, 56% of the participants in our sample were female. The median age was 26–35 years for both male and female participants. However, with 1901 observations, the largest frequency was in the age group of adolescents and young adults (15–25 years). About 76.94% of the participants were African, while Indians were the smallest group in the sample.

The average BMI of women in our study was 28.81 kg/m^2^ and for men 23.70 kg/m^2^. About 43% of the respondents were categorized with a normal weight, while around 8% were underweight, around 23% were overweight and around 27% were obese, as seen in Table 2.

Of the obese participants, 1518 were female, and 371 were male. The assessment of the dietary intake of the participants was based on data for 6325 participants, of which more than half of the male (55.32%) and female (55.23%) reported an unhealthy daily diet. The physical inactivity of the participants was measured with the help of a submaximal cardiovascular fitness test, which took place during the clinical examination of the respondents. Only 2539 respondents participated in the fitness test. Of those, about 30% of the men and about 25% of the women were classified as unfit. Regarding smoking behavior, 82% of the participants indicated to be a non-smoker. Of the smokers, 69% were male.

### 3.1. Concentration Indices

The CI provides information on health inequalities between participants with different SES. Table 3 gives an overview of the weighted CIs of ‘obesity’, ‘unhealthy diet’, and ‘physical inactivity’ for the total sample and by gender. The CIs of ‘obesity’ and ‘physical inactivity’ was positive, which indicates a concentration of obesity and the tendency to be physically inactive among the more affluent population. The index values by gender further showed that these inequalities were larger among males than among females. The results of physically inactive females were found to be not statistically significant. The index value of ‘smoking’ was close to zero, which indicated that in total, no prevalent inequalities exist. The index values of the variable ‘unhealthy diet’ and ‘smoking males’ were negative, which indicated a concentration of unhealthy diet among the poor and a concentration of smoking among the poor males. For ‘unhealthy diet’, there were no considerable differences between male and female participants.

### 3.2. Multivariate Analysis

Table 4 presents the results of the multivariate analysis. For the ‘obese individuals’ model, all age groups (compared to the reference age group 15–25), employment status ‘employed’ and wealth index were significant and positively associated with obesity, while the male gender, the Indian race (compared to the reference race group African) and ‘smoking’ were statistically significant but negatively associated with obesity. The increased risk of obesity among employed individuals is in line with the higher risk of obesity among wealthier individuals.

The results for ‘obese females’ showed a positive and statistically significant association of obesity for all age groups (compared to the reference age group 15–25). This result was highest for age groups 46–55 and 56–65. A negative association with the education status of ‘no schooling’ was found (compared to the reference group of secondary education). This differed from the results for ‘obese males’, as only the age groups 46–55 and 56–65 were statistically significant and positively associated with obesity (compared to the reference age group 15–25) while the Indian race (compared to the reference race group African) and unhealthy diet were negatively associated with obesity. The factor ‘smoking’ decreased the risk of obesity among men significantly.

### 3.3. Decomposition Analysis

Table 4 shows the results of the decomposition analysis. Next to the wealth index, other main contributors to the inequalities in obesity were the age groups ‘46–55’ and ‘56–65’, ‘primary education’, the employment status ‘employed’ as well as the marital status ‘married’.

The inequalities were more distinct among obese males, but more women were obese than men. Men were 11.32% less likely to be obese than women. The distribution of obese women over the wealth quintiles showed that the distribution of obese females was almost even across the study sample, with a tendency to the wealthier groups.

The results of the decomposition analysis in Table 5 further showed that women in the age groups 26–35 and 46–55 were up to 6% more likely to be obese than adolescent or young adult women in the reference age group 15–25. Other main contributors to inequalities in obesity among women were the employment status ‘employed’, marriage status ‘married’ as well as ‘no schooling’ or ‘primary education’ and the wealth index. The African race was the most prevalent race among obese women. The Caucasian race (−25.80%) and tertiary education (−32.91%) decreased the inequalities in female obesity.

For males, the Caucasian and Colored race category, as well as the factor of ‘tertiary education’ increased the inequalities in obesity. Additionally, for males, a tendency of larger inequalities in obesity was found for the factors ‘married’, ‘employed’, and for the age group 46–65.

Since we had a limited number of respondents who agreed to participate in the cardiovascular fitness test, the explanatory value of the variable of physical inactivity could be questioned. To check whether our results are robust, we performed all analyses without controlling for physical activity. However, this did not alter the effect size substantially. We, thus, decided to keep the variable in the analysis.

## 4. Discussion

This analysis showed that pro-rich inequalities in association with obesity exist in South Africa. The inequalities among obese men were larger (CI of 0.16) than among women (CI of 0.09). However, more women were obese in total.

The main factors associated with obesity among adolescents, young adults, and adults were examined with the help of the GLM (multivariate analysis). This showed that people of Indian origin and smoking individuals are less likely to be obese. Smoking decreases the risk of obesity among men significantly, while marriage seems to increase the risk of obesity for women and men.

The African race was found to be the most prevalent race among obese women, although other races make up a high proportion as well. The associations between race and obesity risk can be linked to cultural differences, as well as the difference in nutrition but the perception of beauty, health and body images, too [20,38,39,40,41,42]. The main contributor to the inequalities in obesity is the wealth index.

Our study confirms the findings of McLaren [22], who showed that in low- and middle-income countries, obesity is more prevalent among the rich and also the findings by Alaba and Chola [21], who showed the same relation between a higher SES and obesity in South Africa. Alaba and Chola [21] also found larger inequalities in men (CI of 0.28) than in women (CI of 0.11) in South Africa.

Similar to our results, Puoane et al. [28] and WHO [1] also find a higher prevalence of obesity among women, which could be explained by different factors. There is evidence in former studies that married women are more likely to be obese, which is in line with the findings in this study for married individuals [43]. Another possible explanation could be the perception of a woman’s body type or size. There is evidence from studies in Senegal and Uganda that women with an overweight or obese figure are considered more attractive and healthier, as well as being associated with positive personality traits [43]. Alaba and Chola [21] found this to be a large contributor to obesity among the population of the African race. A 2010 study in Nigeria showed that obesity is socially and culturally accepted [44] and Mokhtar et al. [45] found obesity in the African culture to be “…a cultural symbol of beauty, fertility and prosperity” [31]. Shisana et al. [30] show that only 10% of men and 14% of women included in the SANHANES survey were able to identify a ‘normal’ bodyweight image based on presented body image silhouettes, and over 76% of the respondents identified themselves as having a ‘fat’ body image based on the silhouettes presented. Yet about 69% of males and 63.3% of females were happy with their current weight [30]. A plausible conclusion here is that individuals accept being overweight or at least do not have a problem with it.

Obesity is more of a problem during pregnancy. An Australian study shows that maternal obesity can cause obstetric and medical complications during childbirth and is associated with social disadvantages [46]. However, it also influences children’s life as maternal obesity increases the risk of childhood obesity, which increases the risk of cardiovascular diseases later in adult life [47].

The increasing obesity rates and inequalities in obesity among adolescents, young adults, and adults in South Africa could be explained by various reasons. Previous studies have mentioned the nutrition transition in South Africa’s population. Specifically, Dinsa, Goryakin, Fumagalli, and Suhrcke [48] explain the pro-rich inequalities with the ability of the more affluent population to consume a surplus of food, while the poor are more likely to face a food shortage. Additionally, the larger inequalities in obese men could be linked to the economic growth of South Africa’s economy, which led to higher employment [21]. This is shown in this analysis as well, as ‘employment’ increases the inequalities in obesity. Furthermore, it was shown that men with a lower SES often carry out work that includes physical activity [21,49], but a shift in the overall economic activity could lead to lower demand for physical activity by workers [50].

As described above, Case and Menendez [43] had similar results on the unfavorable influence of being married on the obesity risk. This was found in a US study of 2016 as well, as they found that if a spouse became obese, the likelihood of the partner becoming obese increased [51]. Cobb et al. [51] explained this with the degree of similar behavior, as couples who are not discordant are more likely to influence each other, also on their weight status. Another possible explanation could be that more women are obese than men and women as caregivers are mainly in charge of the food preparation for the entire family [21]. An individual’s body weight is a combination of numerous different decisions about nutrition, exercise, and other factors. Spouses tend to make these decisions together, therefore strongly influence each other on their weight and overall health status [52]. The decreasing risk to be obese among smokers was found by Cois and Day [53] as well since smoking in their study was significantly associated with lower rates of increasing BMI’s.

The main contributor to the inequalities in obesity is wealth. This is in line with the findings of Alaba and Chola [9], who ascertained that assets contribute the most to inequalities in South Africa (71.4% for women, 24% for men). Similar findings were found in studies that examine the relationship between gross national income or the human development index and obesity [48]. Dinsa et al. [48] mentioned the problem of food shortages among the less affluent population in developing countries. There is evidence from a study in South Asia that energy-dense food is often cheap but nutrient-poor, which might lead to an overall unhealthy diet of less well-off populations [54]. A nutritional transition, with increased intake of energy-dense processed foods and decreased physical activity, was found in Sub-Saharan Africa as well, especially in low-resource settings [55].

The findings of this study that tertiary education increases the obesity risk of adults, in general, was found by Cois and Day [53] as well. This can be due to the fact that tertiary education is a strong indicator of a higher SES and obesity is more prevalent among the rich. The inequalities in obesity are higher for people with low education, which is in line with previous studies, as a low education tends to be an indicator of a lower SES. Accordingly, a tertiary education could decrease inequalities in female obesity by about 33%. Similar results were found in other African countries. Higher education was found to have a decreasing effect on obesity among adolescents, young adults, and adults. An interpretation here could be the advanced skills to read, to use, and interpret health-related information and the overall decision-making abilities about the health, nutrition and lifestyle behavior of individuals with a higher education level [56,57].

In this study, we find that inequalities associated with obesity occur in the age groups 46–55 and 56–65, especially among women. An explanation for this could be that the individuals in these age groups were of school age during the Apartheid regime in South Africa and, therefore, they were affected by the educational system under Apartheid. Caucasian people during Apartheid received a far better education than Africans. Strict segregation between ‘black’ and ‘Caucasian’ schools and insufficient funding for ‘black’ schools and a lack of qualified teachers led to poor pass rates in school and early dropouts [58]. This could have led to less secondary and tertiary education in these age groups. In our study, ‘no schooling’ or ‘primary education’ was shown to increase the inequalities. However, if this holds true one would expect the effect to differ between middle-aged individuals with higher or lower education. As we control for education and ethnic background, we see this effect more likely to be one of becoming middle-aged.

Our study had several strengths and limitations that need to be acknowledged. The use of a questionnaire in addition to a clinical examination was a significant strength of the data alongside the large sample size. In particular, the combination of the two data collection modes increased the reliability of the data. Self-reported data on fitness, weight, height, waist and hip circumference, blood pressure, and pulse rate are seen to be less accurate and even not reliable [30,59], which was avoided in this study as these data were gathered in a medical examination by a professional. We face a low response rate in the clinical examination of especially Caucasian participants and participants in the age group 26–35. The Caucasian race is more prevalent in the affluent population (CI of 0.74, Table 5), and the age group 26–35 is more prevalent in the less affluent population (CI of −0.017, Table 5). This could lead to an underrepresentation of a more affluent part of society and, thus, distort our wealth index. To diminish this bias, we apply sample weights in this study. We found no significant relation between obesity and behavioral factors like physical inactivity or the diet of the individual. The lack of significance could be related to the limited number of respondents who agreed to participate in the cardiovascular fitness test as part of the clinical examination of SANHANES-1, which led to a small sample size. Although we used weighted data in all analyses, it is still questionable how much explanatory value the variable physical inactivity had for obesity within this study. It could be argued that individuals who agreed to participate in the fitness test are more physically active in general. Similar limitations counted for the variable of ‘unhealthy diet’. Although the WHO definition for a healthy diet was applied, the data on a healthy diet were self-reported data and, therefore, less reliable [31,59].

Our study included adolescents, young adults, and adults. The inclusion of the group of adolescents and young adults is especially important since it includes individuals of developmental age, for whom obesity has considerable physical and mental importance. We, however, did not include the group of children (0–14 years old) since they were not included in our data set. Given the growing importance of obesity in South Africa and worldwide, future studies need to specifically focus on the socioeconomic inequalities and obesity among children, adolescents and young adults, which might require a different study approach than that applied to adults.

In numerous studies, the significance and importance of the individuals’ living situation on the obesity risk were found, especially if the individual lives in a rural or urban area. Individuals from rural areas were less likely to be obese [53], but the inequalities in adult obesity tend to be higher in rural areas [21]. The SANHANES-1 questionnaire is missing a question to distinguish whether the individual lives in an area to be defined as rural or urban and, therefore, this information is missing in this study.

## 5. Conclusions

These results show that pro-rich inequalities associated with obesity exist especially among men, while the inequalities among women are smaller. In total, more women than men are obese in South Africa and the obesity risk among women increases with age and differs by race and marital status. The socioeconomic inequalities are mainly explained by wealth, but through age and educational status as well. The most prevalent finding here is that a tertiary education among women decreases inequalities by about 33%.

With regard to policy, education, particularly higher education, plays a key role, especially among women. Not only does investment into education have significant potential in reducing inequalities in obesity, but could also lead to the reduction of other inequalities caused by the political past in the South African society. Especially targeting women with unhealthy body weight is of importance to reduce inequalities within the population, but it is also important for future populations as well. South African women are at high risk of obesity, with numbers increasing. The severity of this manifests itself not only in the negative health effects on the woman herself but on the fetus during pregnancy and the child’s future life as well.

The discussion of the findings of this study underlined the importance of the understanding and sensitivity towards cultural influences on healthy body images and perceptions, especially among women. To achieve the desired outcomes, more tangible measures are needed, such as assuring access to healthy nutrition and offering opportunities to exercise in convenient settings, as well as regulations on the advertisement of unhealthy foods. Individuals need to start perceiving an obese body size as a threat to their health. Therefore, we recommend implementing culture-sensitive interventions to influence social perceptions on healthy body size. This could help to combat rising numbers of obesity and its consequences.

## Figures and Tables

**Table 1:** Descriptive variables.

Variables	Male (*n* = 3126) *n* = Valid Percentage	Female (*n* = 3942) *n* = Valid Percentage	Total (*n* = 7068)
	Male	Female	Total
Age groups			
15–25	923 (30.79%)	978 (25.49%)	1901 (27.81%)
26–35	709 (23.65%)	984 (25.63%)	1693 (24.76%)
36–45	504 (16.82%)	843 (21.97%)	1347 (19.71%)
46–55	410 (13.66%)	484 (12.62%)	894 (13.08%)
56–65	272 (9.07%)	299 (7.8%)	571 (8.36%)
<65	180 (6.02%)	249 (6.48%)	429 (6.28%)
Missing	128	105	233
Race			
African	2342 (74.92%)	3096 (78.54%)	5438 (76.94%)
Caucasian	380 (12.16%)	346 (8.77%)	726 (10.27%)
Coloured	305 (9.76%)	371 (9.42%)	676 (9.57%)
Indian	99 (3.17%)	129 (3.26%)	228 (3.22%)
Education			
No schooling	145 (5.41%)	229 (6.82%)	374 (6.19%)
Primary education	505 (18.9%)	584 (17.43%)	1089 (18.08%)
Secondary education	1728 (64.64%)	2195 (65.47%)	3923 (65.1%)
Tertiary education	295 (11.06%)	344 (10.27%)	639 (10.62%)
Missing	453	590	1043
Employment status			
Economically inactive	924 (35.16%)	1127 (33.92%)	2051 (34.47%)
Employed	965 (36.76%)	861 (25.9%)	1826 (30.69%)
Unemployed	738 (28.08%)	1336 (40.19%)	2074 (34.84%)
Missing	499	618	1117
Civil status			
Married/living together	1144 (44.59%)	1392 (42.08%)	2536 (43.18%)
Never married	1320 (51.46%)	1558 (47.09%)	2878 (49%)
Widowed	61 (2.37%)	256 (7.73%)	317 (5.39%)
Separated/divorced	41 (1.58%)	103 (3.1%)	144 (2.43%)
Missing	560	633	1193

**Table 2:** Weight rates (WHO classification).

Weight Categories	Frequency	Percentage
Underweight	531.84	7.52
Normal Weight	3048.45	43.13
Overweight	1599.31	22.63
Obese	1888.4	26.72
Total	7068	100

**Table 3:** Concentration indices.

Variables	Number of Observations	Concentration Index Value	Std. Error	*p*-Value	Statistically Significant (Yes/No)
Obesity	5824	0.11237036	0.02516368	0.0000	Yes
Obese females	3773	0.08771286	0.03924991	0.0260	Yes
Obese males	2051	0.16266911	0.04839477	0.0009	Yes
Unhealthy diet	5190	−0.11462672	0.0477799	0.0169	Yes
Unhealthy diet females	3384	−0.1003759	0.04581438	0.0290	Yes
Unhealthy diet males	1806	−0.1307734	0.06246661	0.0370	Yes
Physical inactivity	2120	0.1312212	0.0481173	0.0067	Yes
Physical inactive females	1333	0.06373424	0.04484888	0.1562	No
Physical inactive males	787	0.20552333	0.07567503	0.0070	Yes
Smoking	5262	0.01902339	0.02657773	0.4745	No
Smoking females	3430	0.06639417	0.03142297	0.0352	Yes
Smoking males	1832	−0.05967833	0.04804218	0.2149	No

**Table 4:** GLM-multivariate analysis of CI.

Variables	Obese Individuals Number of Observations: 3704 Log Pseudolikelihood: −9,144,832.25 AIC: 4937.827 BIC: 1.83 × 10^7^			Obese Females Number of Observations: 2455 Log Pseudolikelihood: −6,398,868.68 AIC: 5212.946 BIC: 1.28 × 10^7^			Obese Males Number of Observations: 1249 Log Pseudolikelihood: −2,431,639.17 AIC: 3893.773 BIC: 4,854,530		
	Coefficient	Std. Error	*p*-Value	Coefficient	Std. Error	*p*-Value	Coefficient	Std. Error	*p*-Value
Male	−0.91698	0.11150	0.000	-	-	-	-	-	-
Age 26–35	0.50064	0.11438	0.000	0.71144	0.12546	0.000	−0.14585	0.24923	0.558
Age 36–45	0.65567	0.13842	0.000	0.88392	0.14170	0.000	0.19804	0.28741	0.491
Age 46–55	1.17429	0.18282	0.000	1.274706	0.16051	0.000	0.61427	0.31058	0.048
Age 56–65	1.10441	0.17595	0.000	1.191559	0.18514	0.000	0.90766	0.31314	0.004
Age >65	0.57404	0.19605	0.003	0.84492	0.21157	0.000	−0.12981	0.39960	0.745
Race Caucasian	0.05260	0.26372	0.842	−0.21452	0.30721	0.485	0.26803	0.33407	0.422
Race Colored	−0.06405	0.10880	0.556	−0.22971	0.13715	0.094	0.17067	0.20032	0.394
Race Indian	−0.47985	0.20853	0.021	−0.41052	0.25989	0.114	−0.83754	0.29185	0.004
No schooling	−0.18387	0.12792	0.151	−0.30182	0.15195	0.047	0.03781	0.23036	0.870
Primary education	−0.16453	0.08576	0.055	−0.13009	0.09579	0.174	−0.20981	0.18549	0.258
Tertiary education	−0.18946	0.22722	0.404	−0.37707	0.22104	0.088	0.13418	0.28658	0.640
Employed	0.24203	0.10989	0.028	0.18344	0.12297	0.136	0.40875	0.21542	0.058
Unemployed	0.12969	0.09212	0.159	0.09961	0.09931	0.316	0.17006	0.22575	0.451
Married	0.14127	0.08591	0.100	0.14610	0.09908	0.140	0.21179	0.19585	0.280
Widowed	0.11170	0.13225	0.398	0.10750	0.14500	0.458	−0.05412	0.48659	0.911
Separated/divorced	0.05517	0.26203	0.833	0.02801	0.27347	0.918	0.30826	0.52699	0.559
Smoking	−0.40323	0.14377	0.005	−0.19652	0.21285	0.356	−0.45466	0.18111	0.012
No observation smoking	0.05031	0.10648	0.637	0.00361	0.11516	0.975	0.16730	0.19700	0.396
Physical inactive	−0.01194	0.13801	0.931	−0.09017	0.15554	0.562	0.12400	0.28434	0.663
Unhealthy diet	−0.00439	0.08273	0.958	0.17543	0.09220	0.057	−0.44569	0.16006	0.005
Wealth index	0.10616	0.04967	0.033	0.06611	0.05761	0.251	0.16985	0.09500	0.074
Constant	−0.91679	0.11751	0.000	−1.061186	0.13235	0.000	−1.56924	0.22855	0.000

**Table 5:** Decomposition of socioeconomic inequalities in obesity.

		Obese Individuals		Females		Males	
Variables	Concentration Index	Elasticity	Contribution in %	Elasticity	Contribution in %	Elasticity	Contribution in %
Male	0.021	−0.1132	−8.4574	-	-	-	-
Age 26–35	−0.0165	0.0346	−2.0264	0.061	−4.5736	−0.0061	0.2482
Age 36–45	0.0213	0.0361	2.7304	0.0603	5.8461	0.0066	0.3468
Age 46–55	0.0624	0.0429	9.5255	0.0577	16.4226	0.0136	2.0951
Age 56–65	0.0845	0.0258	7.754	0.0345	13.2872	0.0129	2.6795
Age >65	−0.0223	0.0101	−0.7974	0.0183	−1.8641	−0.0014	0.0758
Race Caucasian	0.7422	0.0015	3.9834	−0.0076	−25.8044	0.0047	8.5354
Race Colored	0.304	−0.0017	−1.8511	−0.0076	−10.5442	0.0028	2.074
Race Indian	0.4139	−0.0043	−6.355	−0.0046	−8.6351	−0.0046	−4.6639
No schooling	−0.4025	−0.0032	4.5544	−0.0065	11.874	0.0004	−0.3938
Primary education	−0.2592	−0.0083	7.6609	−0.0081	9.6208	−0.0064	4.1078
Tertiary education	0.5208	−0.0056	−10.4106	−0.0139	−32.9088	0.0024	3.1002
Employed	0.1694	0.0207	12.5046	0.0195	15.0528	0.0213	8.8794
Unemployed	−0.1159	0.0126	−5.205	0.012	−6.349	0.0101	−2.8697
Married	0.1328	0.017	8.0502	0.0218	13.2221	0.0155	5.0743
Widowed	−0.1358	0.0017	−0.8123	0.002	−1.2416	−0.0005	0.1655
Separated/divorced	0.016	0.0004	0.0213	0.0002	0.0172	0.0013	0.0501
Smoking	0.0279	−0.0204	−2.024	−0.0123	−1.5667	−0.014	−0.9596
No observation smoking	−0.0039	0.0078	−0.109	0.0007	−0.0124	0.0157	−0.1524
Physical inactive	0.1212	−0.0004	−0.1729	−0.0038	−2.0746	0.0025	0.7553
Unhealthy diet	−0.0532	−0.0007	0.1286	0.0337	−8.1568	−0.042	5.486
Wealth index	−6.5633 ^a^	−0.0024	56.7729	−0.0019	56.1544	−0.0024	38.1908
Total			75.4651		37.7659		72.8248

^a^ Not between −1 and 1 as the wealth index was used as the rank variable.

## Data Availability

Restrictions apply to the availability of these data. Data was obtained from the Human Science Research Council in Pretoria, South Africa and are available upon request at http://hdl.handle.net/20.500.11910/2864 (last accessed on 27 September 2021) with the permission of Human Science Research Council in Pretoria, South Africa.
